# Value-Based State-Directed Payments in Medicaid Managed Care

**DOI:** 10.1001/jamahealthforum.2025.1666

**Published:** 2025-06-20

**Authors:** Max Yates, Jonathan Gonzalez-Smith, Kun Li, Asher Wang, Robert R. Saunders

**Affiliations:** 1 Harvard Medical School, Boston, Massachusetts; 2 Duke-Margolis Institute for Health Policy, Duke University, Washington, DC; 3 Center for Medicare and Medicaid Innovation, Washington, DC; 4 Medicaid and CHIP Payment and Access Commission, Washington, DC

## Abstract

This cross-sectional study examined the scale and scope of value-based state-directed payments in Medicaid managed care.

## Introduction

Accelerating value-based payments (VBPs) in Medicaid is a priority for the Centers for Medicare & Medicaid Services (CMS).^1^ Historically, states had limited authority to direct payments in Medicaid managed care. State-directed payments (SDPs), introduced in 2016, granted states additional authority to determine how managed care organizations (MCOs) reimburse providers (defined as clinicians and health care organizations). Through SDPs, states can enhance payments or advance VBP arrangements, such as shared savings. SDP use has grown, with $110 billion in projected expenditures approved between February 2023 and August 2024.^2^

SDPs could provide a lever for states to scale VBP approaches that MCOs may not implement on their own due to financial risk, limited provider engagement, operational complexity, and misaligned incentives. Yet, most states use SDPs to increase provider payments without linking them to quality.^2^ Given limited research on SDPs’ role in advancing VBPs, this study examined the scale and scope of value-based SDPs across states.

## Methods

We analyzed approved value-based SDPs among 430 unique SDPs approved by CMS between February 2023 and May 2024.^3^ Each application detailed the payment type, amount, quality measures used, and providers targeted. In accordance with the Common Rule (45 CFR 46), this cross-sectional study was exempt from review and informed consent because it was not human participant research. The STROBE guideline was followed.

Using the Health Care Payment Learning and Action Network Alternative Payment Model framework,^4^ states can classify value-based SDPs as category 2 (pay for performance or reporting), 3 (shared savings, episodic bundled payments), or 4 (population-based payments) (eTable in Supplement 1). We reported amounts of value-based SDPs by targeted provider type. If no category was reported or the provider classification was unclear, 3 of us (M.Y., J.G.S., K.L.) independently reviewed SDP applications (ie, preprints) to reach a consensus. Statistical analysis was performed with Excel, Office 365 (Microsoft).

## Results

Among 77 value-based SDPs, 69 were category 2 (90%), 4 were category 3 (5%), and 4 were category 4 (5%). Of 42 states eligible to use SDPs, 22 submitted value-based SDPs. Four states submitted advanced VBP arrangements (categories 3 and 4; Figure).

**Figure.**
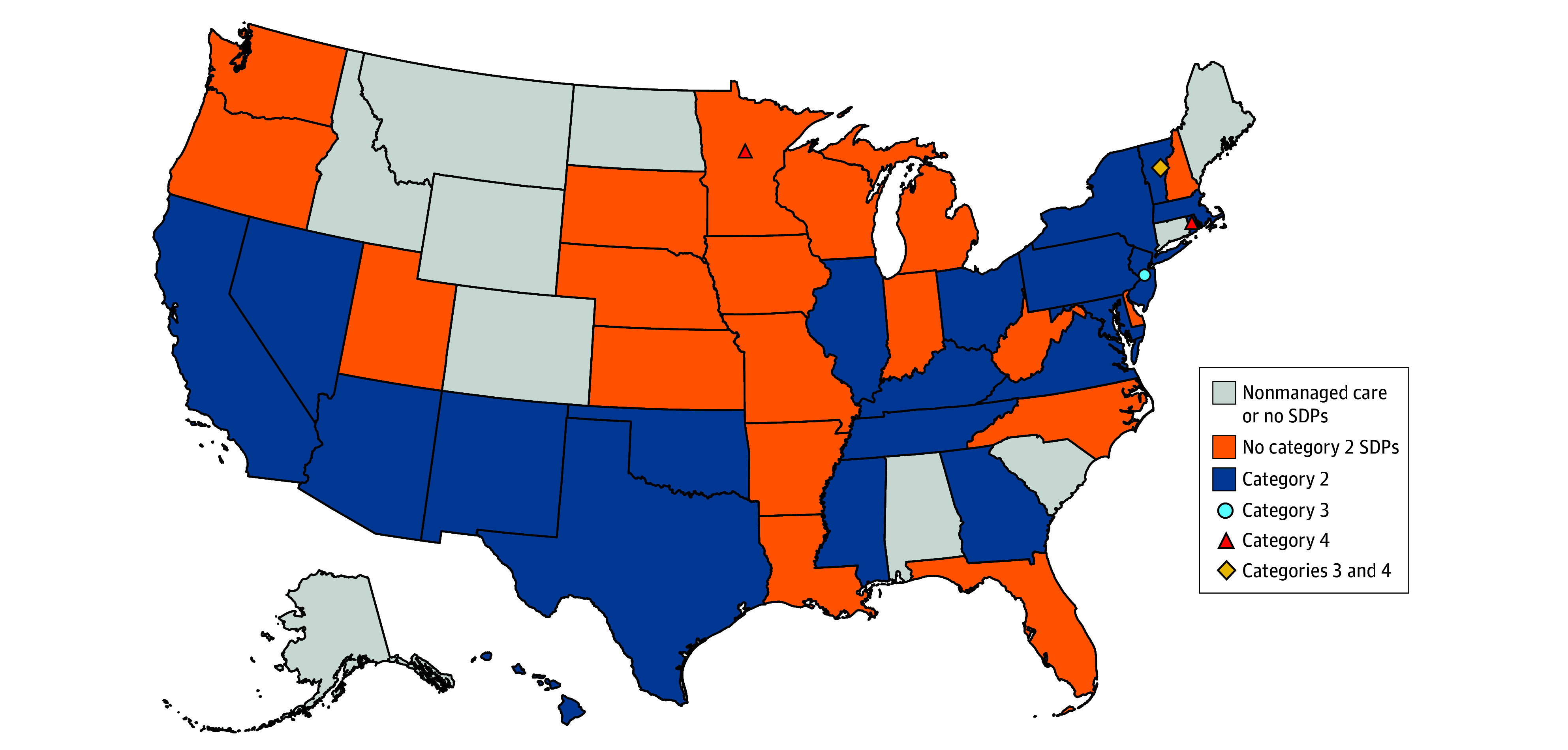
State Adoption of Value-Based Payment in State Directed Payments (SDPs) by Learning and Action Network’s Category of Largest Share of Funds, February 2023 to May 2024 SDP arrangements based on the Health Care Payment Learning and Action Network’s Value-Based Payment Framework include category 2 (pay for performance or reporting), category 3 (shared savings accountable care organization, episodic bundled payments), and category 4 (population-based payments). States that submitted SDPs for provider payment increases and not value-based payments are shown in orange, except for Minnesota, which submitted provider payment increases and value-based payment SDPs, the latter of which were only category 4 payments.

Value-based SDPs totaled $7.8 billion, accounting for approximately 5% of total SDP spending ($144.3 billion) during this period. Hospitals (academic and nonacademic) accounted for 71% of the $7.8 billion value-based SDPs compared with 84% of $144.3 billion in total SDP spending, followed by 13% to nursing facilities (11% of total SDP spending), 11% to Medicaid Accountable Care Organizations (<1%), 4% to behavioral health providers (16%), and less than 1% to primary care (9%). Although primary care and behavioral health represented a small share of total payments, they were targeted in 7 and 9 states, respectively (Table). Only $12 million and $818 million of value-based SDPs were category 3 and 4, respectively.

**Table.** Value-Based SDPs by LAN Category and State

State	Sum of VBP portion of SDP on application ($, million)			
	LAN category			Total
	2	3	4	
Arizona	48.6	NA	NA	48.6
Primary care services	21.1	NA	NA	21.1
Behavioral health services (inpatient/outpatient)	25.8	NA	NA	25.8
Inpatient or outpatient hospitals (nonacademic medical centers)^a^	1.7	NA	NA	1.7
California	2105.4	NA	NA	2105.4
Inpatient or outpatient hospitals (nonacademic medical centers)^a^	2105.4	NA	NA	2105.4
Georgia	46.4	NA	NA	46.4
Inpatient or outpatient hospitals (nonacademic medical centers)^a^	46.4	NA	NA	46.4
Hawaii	97.7	NA	NA	97.7
Nursing facility services	5.3	NA	NA	5.3
Inpatient or outpatient hospitals (nonacademic medical centers)^a^	92.4	NA	NA	92.4
Illinois	2.4	NA	NA	2.4
Primary care services	2.4	NA	NA	2.4
Kentucky	1563.3	NA	NA	1563.3
Academic medical centers	1054.0	NA	NA	1054.0
Inpatient or outpatient hospitals (nonacademic medical centers)^a^	509.3	NA	NA	509.3
Maryland	Unknown^b^	NA	NA	Unknown^b^
Academic medical centers	Unknown^b^	NA	NA	Unknown^b^
Massachusetts	1387.3	NA	NA	1387.3
Behavioral health services (inpatient or outpatient)	232.7	NA	NA	232.7
Inpatient/outpatient hospitals (nonacademic medical centers)^a^	1154.7	NA	NA	1154.7
Minnesota	NA	NA	Unknown^c^	Unknown^c^
Medicaid ACOs	NA	NA	Unknown^c^	Unknown^c^
Mississippi	39.4	NA	NA	39.4
Academic medical centers	39.4	NA	NA	39.4
Nevada	2.5	NA	NA	2.5
Behavioral health services (inpatient or outpatient)	2.5	NA	NA	2.5
New Jersey	273.0	7.0	NA	280.0
Perinatal care episodes spanning multiple care settings	NA	7.0	NA	7.0
Inpatient or outpatient hospitals (nonacademic medical centers)^a^	273.0	NA	NA	273.0
New Mexico	230.6	NA	NA	230.6
Nursing facility services	160.6	NA	NA	160.6
Inpatient or outpatient hospitals (nonacademic medical centers)^a^	70.0	NA	NA	70.0
New York	52.0	NA	NA	52.0
HCBS or personal care services	14.0	NA	NA	14.0
Inpatient or outpatient hospitals (nonacademic medical centers)^a^	38.0	NA	NA	38.0
Ohio	28.1	NA	NA	28.1
Academic medical centers	28.1	NA	NA	28.1
Oklahoma	46.3	NA	NA	46.3
Behavioral health services (inpatient or outpatient)	46.3	NA	NA	46.3
Pennsylvania	175.0	NA	NA	175.0
Nursing facility services	30.0	NA	NA	30.0
Inpatient or outpatient hospitals (nonacademic medical centers)^a^	145.0	NA	NA	145.0
Rhode Island	5.9	NA	159.6	165.5
Primary care services	5.9	NA	NA	5.9
Medicaid ACOs	NA	NA	159.6	159.6
Tennessee	107.8	NA	NA	107.8
HCBS or personal care services	50.0	NA	NA	50.0
Primary care services	53.9	NA	NA	53.9^b^
Academic medical centers	3.9	NA	NA	3.9
Texas	624.6	NA	NA	624.6
Nursing facility services	624.6	NA	NA	624.6
Vermont	17.4	4.9	658.3	680.7
HCBS or personal care services	17.3	4.9		22.3
Behavioral health services (inpatient or outpatient)	0.1	NA	NA	0.1
Medicaid ACOs	NA	NA	658.3	658.3
Virginia	167.4	NA	NA	167.4
Nursing facility services	167.4	NA	NA	167.4
Total	7021.3	11.9	817.9	7851.1

Abbreviations: ACO, accountable care organization; HCBS, home- and community-based service; LAN, Learning and Action Network; NA, not applicable; SDP, state-directed payment; VBP, value-based payment.

^a^ Inpatient or outpatient hospitals include SDP applications that indicate either inpatient or outpatient hospital services as the affected class of providers. SDP arrangements were identified as described in the SDP application: category 2, pay for performance, reporting, and quality bonuses; category 3, episodic bundle payments; category 4, population-based payments.

^b^ The academic medical center preprint in Maryland has “unknown” for the portion of funds to VBP because the SDP application did not contain sufficient information to discern what amount was allotted to VBP compared to fee-for-service.

^c^ Minnesota’s SDP application referenced the attachments for values of the payment arrangement. However, Centers for Medicare & Medicaid Services does not include attachments in the publication of SDP applications, so it was not possible to determine the amount with the information provided.

## Discussion

SDPs offer states a potential mechanism to improve patient outcomes by accelerating VBPs in Medicaid managed care. However, less than 10% of SDPs leveraged advanced VBP arrangements such as bundled payments or population-based payments. Value-based SDPs were predominantly pay-for-performance and typically involved bonus payments to providers that met quality, care coordination, or satisfaction criteria. Such arrangements could have limited implications for improving health outcomes^5^ due to emphasis on process-driven measures rather than patient-centered outcomes.

We also found hospitals were the predominant target of value-based SDPs, which indicates opportunities to engage specialists in VBPs given their historically limited participation.^6^ However, engaging specialists through advanced payment arrangements can be challenging, particularly given Medicaid’s existing challenges in attracting and retaining these providers.

CMS recently revised regulations to offer states more pathways to implement advanced value-based SDP arrangements, supplementing Medicaid MCOs’ existing ability to form value-based contracts directly with providers. Additional CMS actions could accelerate adoption of advanced payment arrangements, such as streamlining approval for SDPs that incorporate category 3 or 4 payments.

The study was limited to a 1-year cross section and relied on state applications, which may not reflect final SDP funding distributions post-approval. Greater transparency from CMS regarding SDP implementation and payment flow could enable a deeper evaluation of their role in advancing value-based care.

## Notes

Supplement 1.
eMethods

Supplement 2.
Data Sharing Statement
