# Supplementary material for: Value-Based State-Directed Payments in Medicaid Managed Care
Source: JAMA Health Forum. 2025 Jun 20;6(6):e251666. doi: 10.1001/jamahealthforum.2025.1666 (PMC12181788; doi:10.1001/jamahealthforum.2025.1666)
Supplement: Supplement 1. — eMethods [file jamahealthforum-e251666-s001.pdf]

Supplemental Online Content

Yates M, Gonzalez-Smith J, Li K, Wang A, Saunders R. Value-based state directed payments in Medicaid managed care. *JAMA Health Forum.* 2025;6(6): e251666. doi: 10.1001/jamahealthforum.2025.1666

eMethods

This supplemental material has been provided by the authors to give readers additional information about their work.

© 2025 Yates M et al. *JAMA Health Forum*

eMethods

Health Care Payment Learning & Action Network Alternative Payment Model Framework

LAN category	Description
1, Fee-for-service, with no link to quality and value	NA
2, Fee-for-service with link to quality and value	Foundational payments for infrastructure and operation
	Pay-for-reporting
	Pay-for-performance
3, Alternative payment models built on fee-for-service architecture	Alternative payment models with shared savings
	Alternative payment models with shared savings and downside risk
	Risk-based payments not linked to quality
4, Population-based payment	Condition-specific population-based payment
	Comprehensive population-based payment
	Integrated finance and delivery system
	Capitated payments not linked to quality

Source: Health Care Payment Learning & Action Network. Alternative Payment Model (APM) Framework. 2017. https://hcp-lan.org/apm-framework/
