# Supplementary material for: Value-Based State-Directed Payments in Medicaid Managed Care
Source: JAMA Health Forum. 2025 Jun 20;6(6):e251666. doi: 10.1001/jamahealthforum.2025.1666 (PMC12181788; doi:10.1001/jamahealthforum.2025.1666)
Supplement: Supplement 2. — Data Sharing Statement [file jamahealthforum-e251666-s002.pdf]

Data Sharing Statement

Yates. Value-Based State-Directed Payments in Medicaid Managed Care. *JAMA Health Forum*. Published June 20, 2025. doi:10.1001/jamahealthforum.2025.1666

Data
Data available: No

Additional Information
Explanation for why data not available: The data are publicly available from the Centers for Medicaid and CHIP Services.
